# Integrated Transcriptomic and Metabolomic Analyses Identify Critical Genes and Metabolites Associated with Seed Vigor of Common Wheat

**DOI:** 10.3390/ijms25010526

**Published:** 2023-12-30

**Authors:** Zhenrong Yang, Weiguo Chen, Tianxiang Jia, Huawei Shi, Daizhen Sun

**Affiliations:** 1 College of Agriculture, Shanxi Agricultural University, Jinzhong 030801, China; yzryhyh@hotmail.com (Z.Y.); jtt735982847@hotmail.com (T.J.); shihuawei999@gmail.com (H.S.); 2 College of Life Sciences, Shanxi Agricultural University, Jinzhong 030801, China; chenweiguo01@hotmail.com

**Keywords:** wheat, seed-vigor, artificial aging, transcriptomics, metabolomics

## Abstract

Seed aging is a common physiological phenomenon during storage which has a great impact on seed quality. An in-depth analysis of the physiological and molecular mechanisms of wheat seed aging is of great significance for cultivating high-vigor wheat varieties. This study reveals the physiological mechanisms of wheat seed aging in two cultivars differing in seed vigor, combining metabolome and transcriptome analyses. Differences between cultivars were examined based on metabolomic differential analysis. Artificial aging had a significant impact on the metabolism of wheat seeds. A total of 7470 (3641 upregulated and 3829 downregulated) DEGs were detected between non-aging HT and LT seeds; however, 10,648 (4506 up and 6142 down) were detected between the two cultivars after aging treatment. Eleven, eight, and four key metabolic-related gene families were identified in the glycolysis/gluconeogenesis and TCA cycle pathways, starch and sucrose metabolism pathways, and galactose metabolism pathways, respectively. In addition, 111 up-regulated transcription factor genes and 85 down-regulated transcription factor genes were identified in the LT 48h group. A total of 548 metabolites were detected across all samples. Cultivar comparisons between the non-aged groups and aged groups revealed 46 (30 upregulated and 16 downregulated) and 62 (38 upregulated and 24 downregulated) DIMs, respectively. Network analysis of the metabolites indicated that glucarate O-phosphoric acid, L-methionine sulfoxide, isocitric acid, and Gln-Gly might be the most crucial DIMs between HT and LT. The main related metabolites were enriched in pathways such as glyoxylate and dicarboxylate metabolism, biosynthesis of secondary metabolites, fatty acid degradation, etc. However, metabolites that exhibited differences between cultivars were mainly enriched in carbon metabolism, the TCA cycle, etc. Through combined metabolome and transcriptome analyses, it was found that artificial aging significantly affected glycolysis/gluconeogenesis, pyruvate metabolism, and glyoxylate and dicarboxylate metabolism, which involved key genes such as *ACS*, *F16P2*, and *PPDK1*. We thus speculate that these genes may be crucial in regulating physiological changes in seeds during artificial aging. In addition, an analysis of cultivar differences identified pathways related to amino acid and polypeptide metabolism, such as cysteine and methionine metabolism, glutathione metabolism, and amino sugar and nucleotide sugar metabolism, involving key genes such as *BCAT3*, *CHI1*, *GAUT1*, and *GAUT4*, which may play pivotal roles in vigor differences between cultivars.

## 1. Introduction

As the world population continues to increase and arable land decreases, high-quality inputs are an important way to improve agricultural productivity [1]. Seed quality plays a crucial role in agricultural production, utilization of genetic resources, biodiversity conservation, and plant community restoration and reconstruction [2,3]. Seed vigor is an important characteristic used to measure seed quality [4]. Generally speaking, seeds with high vigor germinate and emerge more quickly and neatly. They also show strong stress resistance and potential for high yield and quality [5]. However, in practice, due to environmental factors as well as improper storage and physiological changes of the seeds themselves, seeds tend to age, exerting a certain impact on vigor and subsequent seedling growth [6].

Seed aging or deterioration refers to irreversible losses in seeds’ viability, vigor, and germination ability. It is a common phenomenon in the storage process, and usually occurs and develops with the extension of storage time [7]. Seed aging not only is related to agricultural yield and quality, but also has a serious impact on the preservation, utilization, and development of germplasm resources [8]. Seed aging is often accompanied by a series of physiological and biochemical reactions, such as membrane lipid peroxidation [9], soluble sugar and protein degradation [10], gene expression disorder [11], and nucleic acid degradation [12]. At the same time, the membrane system inside the seed is seriously damaged, with increases in permeability [9]. In addition, seeds may show a series of adverse changes, such as slow germination, and low rates or even failure of emergence as the activity of antioxidant enzymes decreases, the content of hydrogen peroxide and reactive oxygen species (ROS) increases, and toxic and harmful substances accumulate [13,14,15,16].

Seed aging may take longer under natural conditions, which causes difficulty in exploring its physiological mechanisms. Therefore, artificial conditions have been used to simulate natural aging processes in an accelerated manner [17,18]. Artificial aging helps to better grasp the changes in the physiological characteristics of seeds during the aging process, providing scientific bases for in-depth research on seed physiology and quality management in practice [19].

High-vigor seeds have greater yield potential than those with low vigor. However, long-term storage leads to a decline in seed vigor. Studies have found that aging is accompanied by the regulation of carbohydrates, lipid metabolism, transcription, and cell division processes in seeds [20,21]. Here, to study the physiological changes and corresponding molecular mechanisms of wheat seed aging, we used artificial aging methods to treat two wheat cultivars with vigor differences (“Zhong 7902” and “Hanxuan 3”), integrating metabolome and transcriptome analyses. This study will provide a theoretical basis for research on wheat seed biology and offer fresh insights into the conservation and utilization of germplasm resources.

## 2. Results

### 2.1. Dynamic Transcriptional Variations in HT and LT Wheat Seeds by Artificial Aging

**Figure 1:**
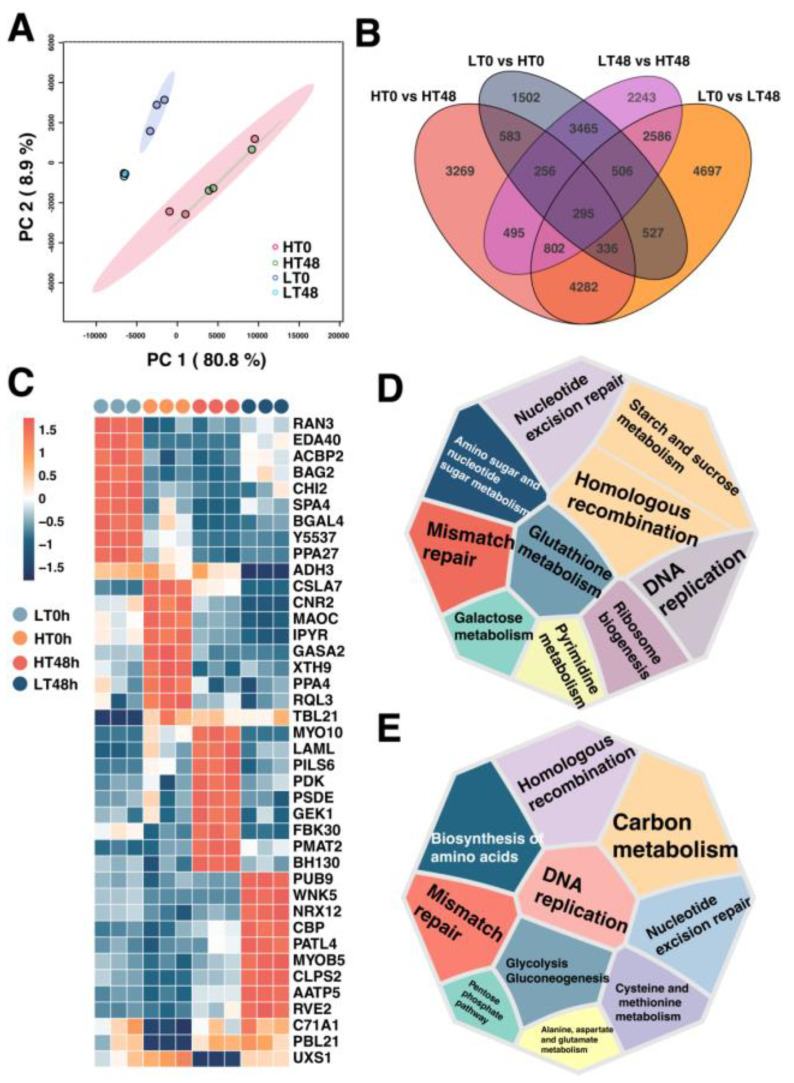
An overview of aged and non-aged seed transcriptomes from both high-vigor (HT) and lowvigor (LT) wheat cultivars. (**A**) Principal component analysis (PCA) of aged and non-aged wheat seed transcriptome from both HT and LT cultivars. The four groups are: HT seeds without aging (HT 0 h), HT seeds aged for 48 h (HT 48 h), LT seeds without aging (LT 0 h), and LT seeds aged for 48 h (LT 48 h). (**B**) Venn diagram of differentially expressed genes (DEGs) between groups. (**C**) Hierarchy clustering of the key DEGs specifically highly expressed in each group. (**D**) Top ten enriched KEGG pathways of DEGs between non-aged groups (HT 0 h versus LT 0 h). (**E**) Top ten enriched KEGG pathways of DEGs between aged groups (HT 48 h versus LT 48 h).

To elucidate distinctions caused by the aging process between HT and LT wheat seeds, a comprehensive global transcriptomic analysis was performed. A total of 121,589 transcripts were detected from all samples, of which 83,770 were defined as validly expressed transcripts after filtering (FPKM > 1). PCA was utilized to assess gene expression levels in each biological replicate (Figure 1A). PC1 proficiently separated HT and LT seeds, while PC2 meticulously represented the impacts of artificial aging treatment on the seeds, with PC1 accounting for 80.8% of total variance and PC2 for 8.9%. This implied that varietal differences had a more substantial impact than aging treatment.

Genes with FDR < 0.05 and FC ≥ 2A were regarded as DEGs. A total of 7470 (3641 upregulated and 3829 downregulated) DEGs were detected between non-aged HT and LT seeds, while 10,648 (4506 upregulated and 6142 downregulated) were detected between the two cultivars after aging treatment (Figure 1B). Differential analysis illustrated that artificial aging enlarged the differences between the two cultivars. Further screening identified key DEGs which were specifically highly expressed between groups. The results showed that genes like *BH130*, *PMAT2*, *FBK30*, *GEK1*, *PSDE*, and *PILS6* might be pivotal for maintaining high vigor in HT seeds during the aging process, whereas genes like *PUB9*, *WNK5*, *NRX12*, *MYOB5*, and *CLPS2* might be crucial in the decline of vigor in LT during aging (Figure 1C). 

Subsequently, KEGG functional enrichment analysis was conducted on the DEGs between HT and LT. DEGs between non-aged groups (HT 0 h versus LT 0 h) were mainly enriched in pathways like nucleotide excision repair, homologous recombination, mismatch repair, DNA replication, galactose metabolism, and starch and sucrose metabolism (Figure 1D), while DEGs between aged seed groups (HT 48 h versus LT 48 h) were mostly related to homologous recombination, nucleotide excision repair, DNA replication, glycolysis/gluconeogenesis, alanine, aspartate and glutamate metabolism, and pentose phosphate pathways (Figure 1E). This may signify that differences in DNA-repair-related genes between HT and LT may result in discrepancies in seed cell activities, thus contributing to the vigor variance between the two varieties. Moreover, transcriptomic analysis indicated that aging treatment may lead to a larger transcriptional difference between the HT and LT seeds, elucidating the progressive nature of anti-aging mechanisms in HT.

### 2.2. Differential Expression of Glycolysis and TCA-Cycle-Related Genes May Affect Wheat Seed Vigor

**Figure 2:**
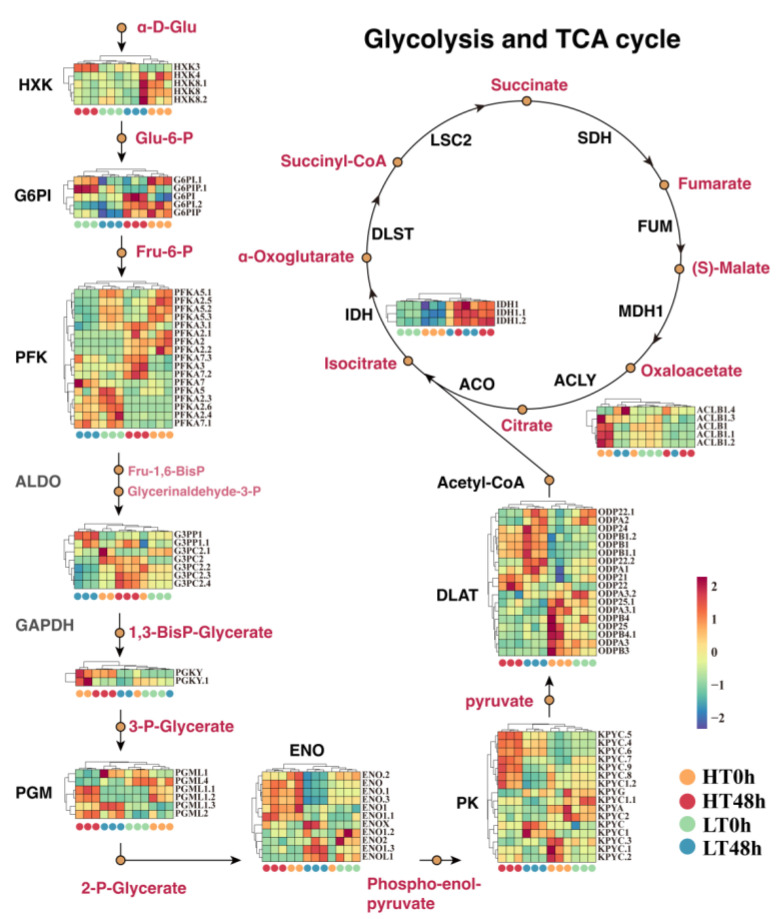
Expression profiling of key gene families in the reconstituted glycolysis and TCA cycle pathway. Eleven key metabolism-related gene families were identified, including HXK (hexokinase), G6PI (glucose-6-phosphate isomerase), PFK (phosphofructokinase), GAPDH (glyceraldehyde-3-phosphate dehydrogenase), PGK (phosphoglycerate kinase), PGM (phosphoglucomutase), ENO (enolase), PK (pyruvate kinase), DLAT (dihydrolipoyl dehydrogenase), IDH (isocitrate dehydrogenase), and ACLY (ATP-citrate synthase).

Functional analysis indicated that the DEGs between aged groups (HT 48 h versus LT 48 h) were significantly enriched in the glycolysis/gluconeogenesis pathway. To evaluate its role in wheat seed aging, we reconstructed the glycolysis/gluconeogenesis and TCA cycle pathway, identifying 11 key metabolism-related gene families, including *HXK* (hexokinase), *G6PI* (glucose-6-phosphate isomerase), *PFK* (phosphofructokinase), *GAPDH* (glyceraldehyde-3-phosphate dehydrogenase), PGK (phosphoglycerate kinase), PGM (phosphoglucomutase), *ENO* (enolase), *PK* (pyruvate kinase), *DLAT* (dihydro lipoyldehydrogenase), *IDH* (isocitrate dehydrogenase), and *ACLY* (ATP-citrate synthase) (Figure 2). The expression patterns of these genes were consistent with RNA-seq and were verified by q-PCR (Supplementary Figure S1).

Five members of the HXK family were detected, among which *HXK4* and *HXK8* exhibited elevated expression in the HT 0 h group, while *HXK3* manifested higher expression in the HT 48 h group. Four G6PI genes, along with eleven PFK genes, exhibited elevated expression in both HT 0 h and HT 48 h. This suggested an enhanced glucose metabolism in HT seeds. Subsequently, four GAPDH genes were also upregulated in HT 48 h. Next, the elevated expression of three PGM genes and six ENO genes in HT 48 h indicated more active glycolysis in HT seeds. Moreover, seven of the PK genes, i.e., *KPYC4, KPYC5, KPYC6, KPYC7, KPYC8,* and *KPYC9*, also displayed elevated expression in HT 48 h, while the DLAT gene family catalyzing the conversion of pyruvate to acetyl-CoA showed elevated expression in LT 48 h and HT 0 h (Figure 2). Lastly, the expression of the IDH genes was elevated in HT 48 h and LT 48 h, while the ACLY family had higher expression in HT 0 h. Overall, HT seeds were found to be more active in the glycolysis/gluconeogenesis pathway, which may allow them to provide more energy to maintain seed vigor during the aging process.

### 2.3. Reduced Expression of Starch Metabolic-Related Genes May Cause Decreased Activity in Wheat Seeds

Functional analysis showed that the DEGs between HT 0 h and LT 0 h were prominently enriched in the starch and sucrose metabolism pathway. We, therefore, reconstructed this pathway to assess its role in wheat seed aging. A total of eight crucial structural gene families were detected: INV (insoluble isoenzyme), HXK (hexokinases), G6PI (glucose-6-phosphate isomerase), PGM (phosphoglucomutase), GLGB (glycogen branching enzyme), UGPA (UDP-Glucose Pyrophosphorylase 2), SUS (sucrose synthase), and AMY (amylase). In this pathway, we noticed similar expression patterns to those of HXK and G6PI in the glycolysis/gluconeogenesis pathway. The expression patterns of these genes were consistent with those of RNA-seq, and this was verified by q-PCR (Supplementary Figure S1).

**Figure 3:**
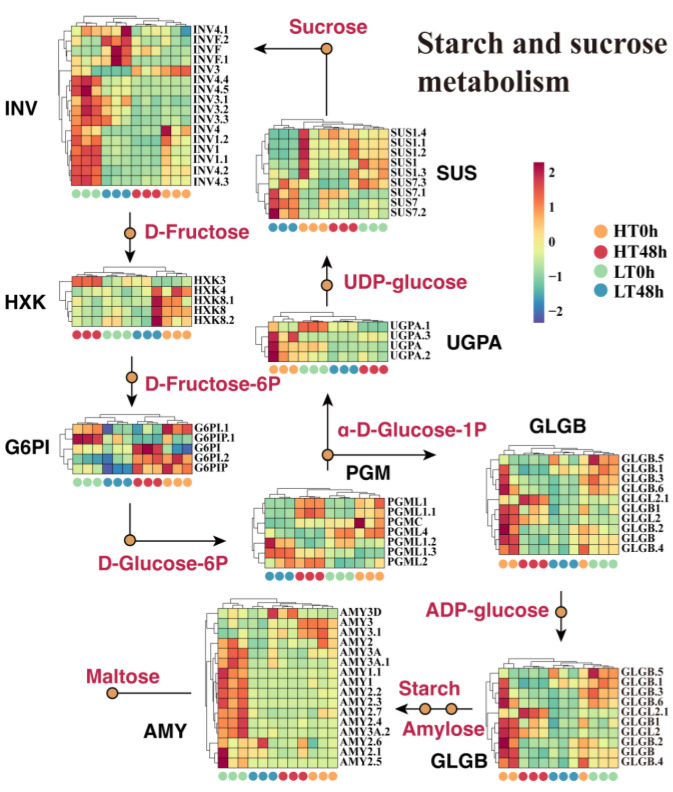
Expression profiling of key gene families in the reconstructed starch and sucrose metabolic pathway. Eight critical structural gene families were identified: INV (insoluble isoenzyme), HXK (hexokinases), G6PI (glucose-6-phosphate isomerase), PGM (phosphoglucomutase), GLGB (glycogen branching enzyme), UGPA (UDP-Glucose Pyrophosphorylase 2), SUS (sucrose synthase), and AMY (amylase).

The results revealed that 11 INV members manifested particularly high expression in LT 0 h (Figure 3). Moreover, nine GLGB genes responsible for starch synthesis, as well as the AMY genes responsible for starch decomposition, also showed high expression in LT 0 h (Figure 3). Additionally, we observed that four PGM genes catalyzing the production of α-D-glucose-1P exhibited elevated expression in HT 48 h. In summary, aged HT seeds displayed more active starch anabolism, whereas non-aged LT seeds exhibited more active starch and sucrose catabolism.

### 2.4. Alterations in the Expression of Galactose Metabolism-Related Genes Resulted in Variations in Wheat Seed Vigor

**Figure 4:**
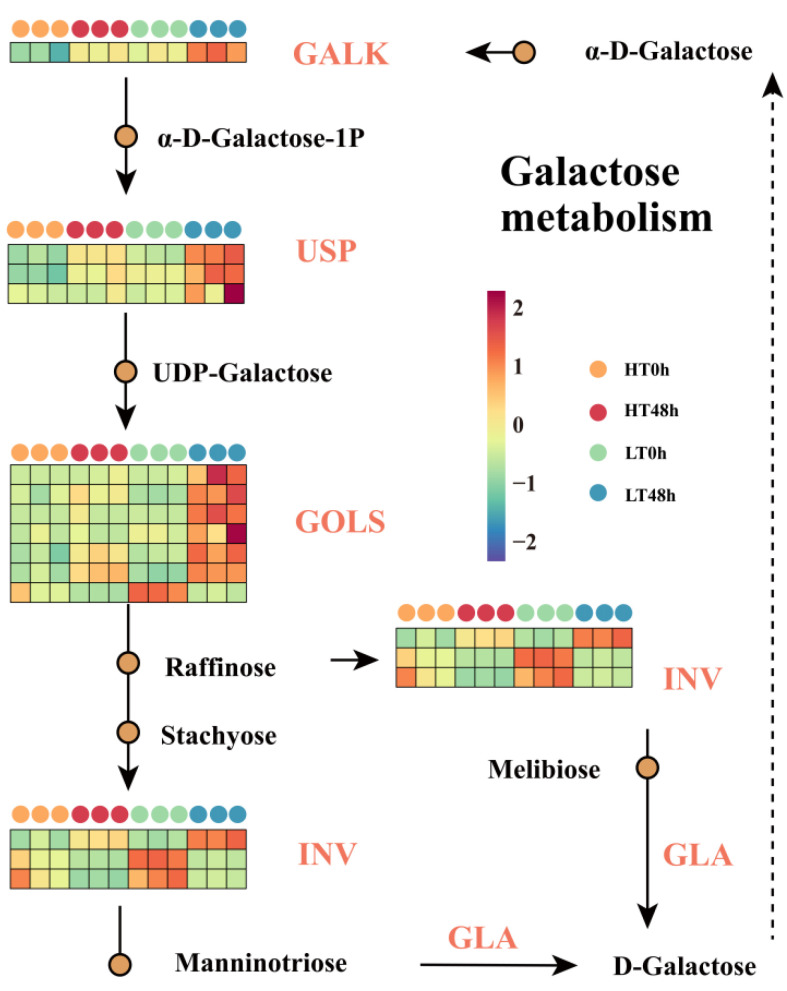
Expression profiling of key genes in the reconstructed galactose metabolic pathway. Four families of critical structural genes were identified: GALK (galactokinase), USP (UDP-sugar pyrophosphorylase), GOLS (galactinol synthase), and INV (insoluble isoenzyme).

Enrichment analysis disclosed that the DEGs between the two cultivars from both aged and non-aged groups were significantly concentrated in the galactose metabolism pathway. We thus reconstructed the galactose metabolism pathway to explore its role during aging (Figure 4). Four critical structural gene families were identified: GALK (galactokinase), USP (UDP-sugar pyrophosphorylase), GOLS (galactinol synthase), and INV (insoluble isoenzyme). The expression patterns of these genes were consistent with that of RNA-seq, and this was verified by q-PCR (Supplementary Figure S1).

Members of the INV family exhibited similar expression patterns to the expression pattern of INV genes, congruent with those of starch and sucrose metabolism. One GALK gene, three USP genes, and six GOLS genes exhibited upregulation in LT 48 h (Figure 4). This indicated that the galactose metabolism pathway was more active in LT wheat seeds, and that it was suppressed in HT wheat seeds. This might suggest that galactose metabolism could be a promoting factor leading to vigor reduction in wheat seeds. 

### 2.5. Dynamic Metabolomic Changes in HT and LT Wheat Seeds by Artificial Aging 

**Figure 5:**
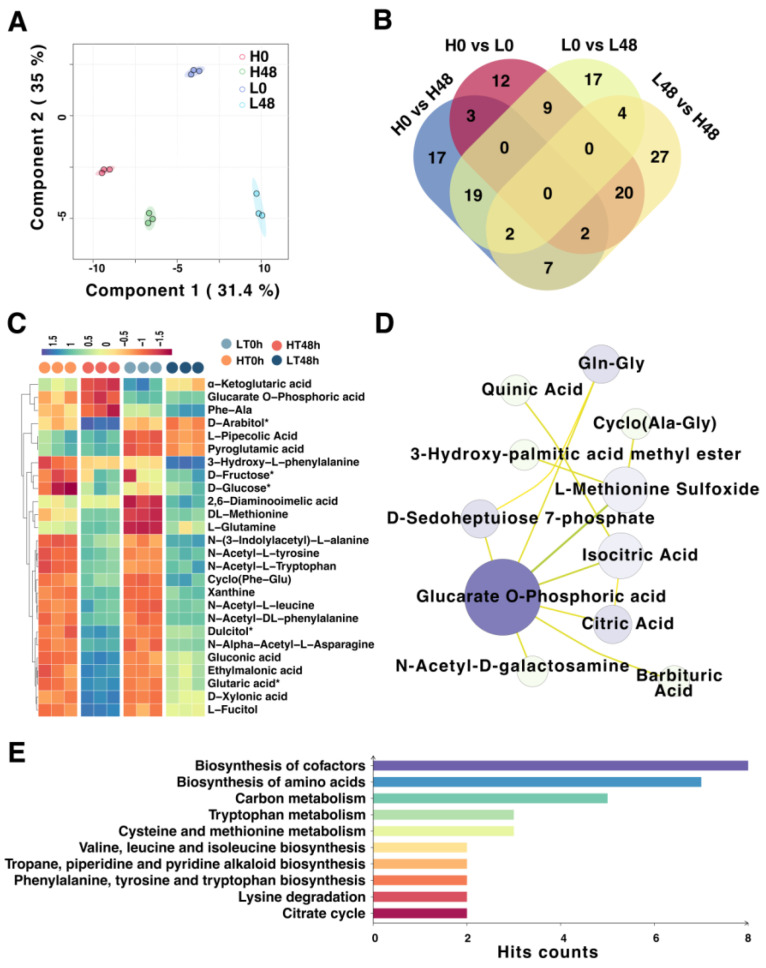
Dynamic metabolomic changes in HT and LT wheat seeds by aging treatment. (**A**) PCA of metabolites in wheat seeds between groups. (**B**) Venn diagram of differential metabolites (DIMs) between groups. (**C**) Hierarchy clustering of the key DIMs specifically overexpressed in different groups. (**D**) Network analysis of DIMs. (**E**) KEGG functional enrichment analysis of DIMs between HT and LT after aging treatment.

To elucidate the disparities between HT and LT wheat seeds via the aging process, we initially assessed the metabolomic variations between aged and non-aged HT and LT seeds. PCA was employed to assess the levels of metabolites (Figure 5A). PC1 effectively distinguished between HT and LT seeds, while PC1 and PC2 together elucidated the impacts of artificial aging on the seeds, with PC1 accounting for 31.4% and PC2 for 35%. This showed a relative congruence between the impacts caused by variety and aging treatment at the metabolomic level.

A total of 548 metabolites were detected across all samples. Metabolites with FDR < 0.05 and VIP ≥ 1 were chosen as differential metabolites (DIMs) for further analysis. Cultivar comparisons between non-aged groups and aged groups revealed 46 (30 upregulated and 16 downregulated) and 62 (38 upregulated and 24 downregulated) DIMs, respectively (Figure 5B). Both HT and LT seeds exhibited more metabolic variations after 48 h of aging treatment than their non-aged counterparts. Key DIMs which were specifically overexpressed were further screened. The results revealed that metabolites such as α−ketoglutaric acid, glucarate O−phosphoric acid, and Phe−Ala may be pivotal for HT seeds to maintain high vigor during aging, whereas 2,6−diaminooimelic acid, DL−methionine, and L−glutamine may be crucial metabolites leading to reduced vigor in LT seeds during aging (Figure 5C).

Subsequent network analysis of the metabolites indicated that glucarate O-phosphoric acid, L-methionine sulfoxide, isocitric acid, and Gln-Gly might be the most crucial DIMs between HT and LT (Figure 5D). KEGG functional enrichment analysis revealed that DIMs between HT 48 h and LT 48 h were primarily concentrated in pathways such as biosynthesis of cofactors; biosynthesis of amino acids; carbon metabolism; tryptophan metabolism; cysteine and methionine metabolism; valine, leucine, and isoleucine biosynthesis; tropane, piperidine, and pyridine alkaloid biosynthesis; phenylalanine, tyrosine, and tryptophan biosynthesis; lysine degradation, and the citrate cycle (Figure 5E). In conclusion, the metabolome and transcriptome analyses exhibited high consistency, and differences in energy metabolism and amino acid metabolism between HT and LT seeds may be crucial factors leading to the variance in vigor.

### 2.6. Association Analysis of Metabolome and Transcriptome Data

To investigate the relationships between the genes and metabolites involved in artificial aging in the two wheat cultivars, DIMs and DEGs of the four comparing groups (LT 0 h versus LT 48 h, HT 0 h versus HT 48 h, LT 0 h versus HT 0 h, and LT 48 h versus HT 48 h) were mapped to the KEGG database. There were 36, 49, 43, and 38 co-mapped pathways in LT 0 h versus LT 48 h, HT 0 h versus HT 48 h, LT 0 h versus HT 0 h, and LT 48 h versus HT 48 h, respectively (Supplementary Table S1). Interestingly, among these co-mapped pathways, fatty acid degradation, propanoate metabolism, glyoxylate and dicarboxylate metabolism, butanoate metabolism, glycolysis/gluconeogenesis, fructose and mannose metabolism, pyruvate metabolism, galactose metabolism, TCA cycle, pentose phosphate pathway, carbon metabolism, and 2−oxocarboxylic acid metabolism were significantly enriched with DIMs and DEGs between LT 0 h and LT 48 h (Supplementary Figure S2A). Meanwhile, pathways such as fructose and mannose metabolism, glycolysis/gluconeogenesis, fatty acid degradation, butanoate metabolism, TCA cycle, starch and sucrose metabolism, propanoate metabolism, galactose metabolism, glyoxylate and dicarboxylate metabolism, carbon metabolism, C5-branched dibasic acid metabolism, pyruvate metabolism, and biosynthesis of secondary metabolites showed significant concentrations when HT 0 h and HT 48 h were compared (Supplementary Figure S2B). We thus detected common pathways enriched by artificial aging, involving a total of seven metabolites (one downregulated and six upregulated) (Supplementary Figure S2C and Table S2), which may have significantly changed during aging and may play key roles in this process. In contrast, DIMs and DEGs between cultivars (LT 0 h versus HT 0 h, and LT 48 h vs HT 48 h) were found to be enriched in pathways such as carbon metabolism, galactose metabolism, cysteine and methionine metabolism, glutathione metabolism, amino sugar and nucleotide sugar metabolism, and biosynthesis of secondary metabolites (Supplementary Figure S2D,E). A number of consistent pathways enriched with DEGs and DIMs between HT and LT wheat seeds were also identified, involving a total of 15 metabolites (4 downregulated and 11 upregulated) (Supplementary Figure S2F and Table S2), which may have contributed to vigor differences between the two cultivars.

### 2.7. Transcript–Metabolite Correlation Network Demonstrating Correlations between DIMs and DEGs

**Figure 6:**
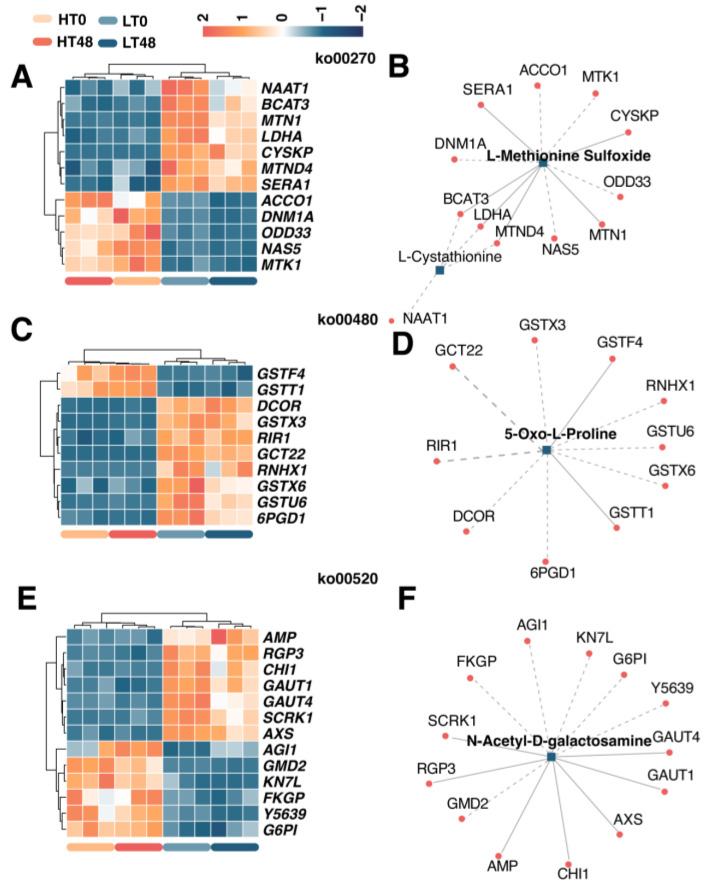
Transcript–metabolite correlation network demonstrating correlations between DIMs and DEGs resulting from varietal differences. (**A**) DEGs related to cysteine and methionine metabolism. (**B**) Correlation network between DIMs and DEGs in cysteine and methionine metabolism. (**C**) DEGs related to glutathione metabolism. (**D**) Correlation network between DIMs and DEGs in the glutathione metabolism pathway. (**E**) DEGs related to amino sugar and nucleotide sugar metabolism. (**F**) Correlation network between DIMs and DEGs in amino sugar and nucleotide sugar metabolism.

To model the synthetic and regulatory characteristics of DIMs and DEGs, subnetworks were constructed to determine transcript–metabolite correlations. Only correlation pairs with correlation coefficients > 0.8 were included in the analysis. DIMs and DEGs caused by varietal differences (LT 0 h versus HT 0 h and LT 48 h versus HT 48 h) were enriched in pathways related to amino acid metabolism and amino sugar metabolism. These pathways included cysteine and methionine metabolism, glutathione metabolism, and amino sugar and nucleotide sugar metabolism (Figure 6). Several classic genes were included in the correlation test, including *BCAT3*, *CHI1*, *GAUT1*, and *GAUT4*. Meanwhile, five pairs showed a positive correlation, and nine pairs were negatively correlated in the network of the cysteine and methionine pathway (Figure 6A,B). Two pairs showed a positive correlation, while eight were negatively correlated in the network of the glutathione metabolism pathway (Figure 6C,D). Seven pairs showed a positive correlation, and six pairs were negatively correlated in the network of the amino sugar and nucleotide sugar metabolism pathway (Figure 6E,F). 

**Figure 7:**
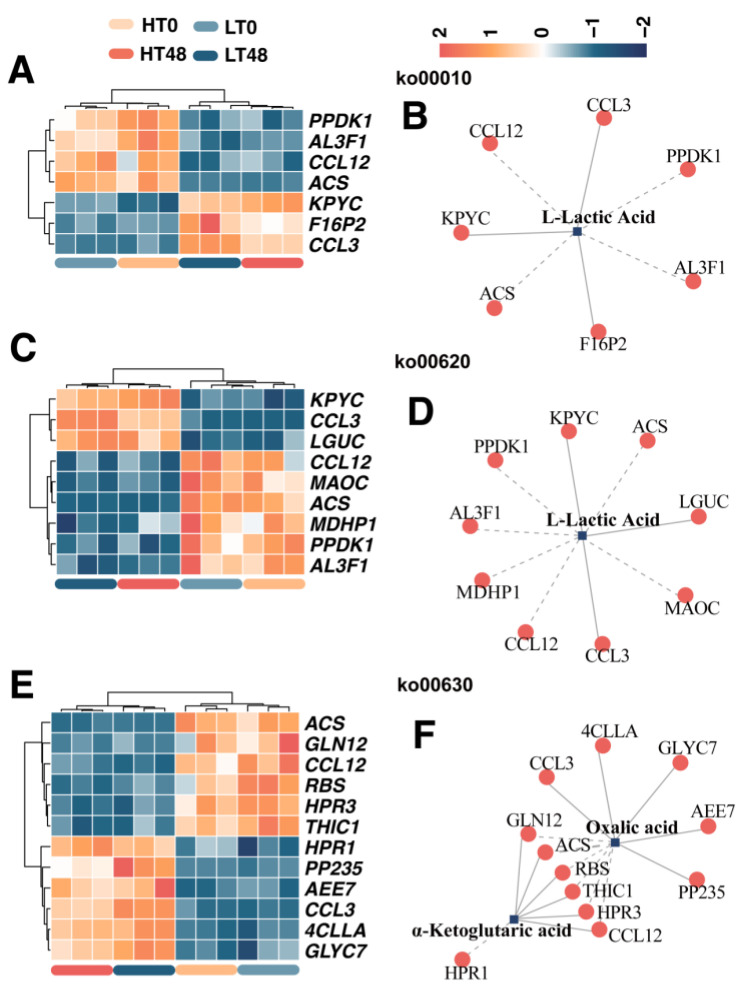
Transcript–metabolite correlation network demonstrating correlations between DIMs and DEGs caused by artificial aging. (**A**) DEGs related to glycolysis/gluconeogenesis. (**B**) Correlation network between DIMs and DEGs in the glycolysis/gluconeogenesis pathway. (**C**) DEGs related to pyruvate metabolism. (**D**) Correlation network between DIMs and DEGs in the pyruvate metabolism pathway. (**E**) DEGs related to glyoxylate and dicarboxylate metabolism. (**F**) Correlation network between DIMs and DEGs in the glyoxylate and dicarboxylate metabolism pathway.

DIMs and DEGs resulting from artificial aging (LT 0 h versus LT 48 h, and HT 0 h versus HT 48 h) were mainly enriched in pathways related to sugar metabolism, acid metabolism, and ketone metabolism. These pathways included glycolysis/gluconeogenesis, pyruvate metabolism, and glyoxylate and dicarboxylate metabolism. Several classic key genes were included in the correlation test, including *ACS*, *F16P2*, *PPDK1*, etc. Meanwhile, three pairs showed a positive correlation, and four pairs were negatively correlated in the network of the glycolysis/gluconeogenesis pathway (Figure 7A,B). Three pairs showed a positive correlation, and six pairs were negatively correlated in the network of the pyruvate metabolism pathway (Figure 7C,D). Ten pairs showed a positive correlation, while seven were negatively correlated in the network of the glyoxylate and dicarboxylate metabolism pathway (Figure 7E,F).

### 2.8. Transcription Factors (TFs) Associated with Aging in Wheat Seeds

**Figure 8:**
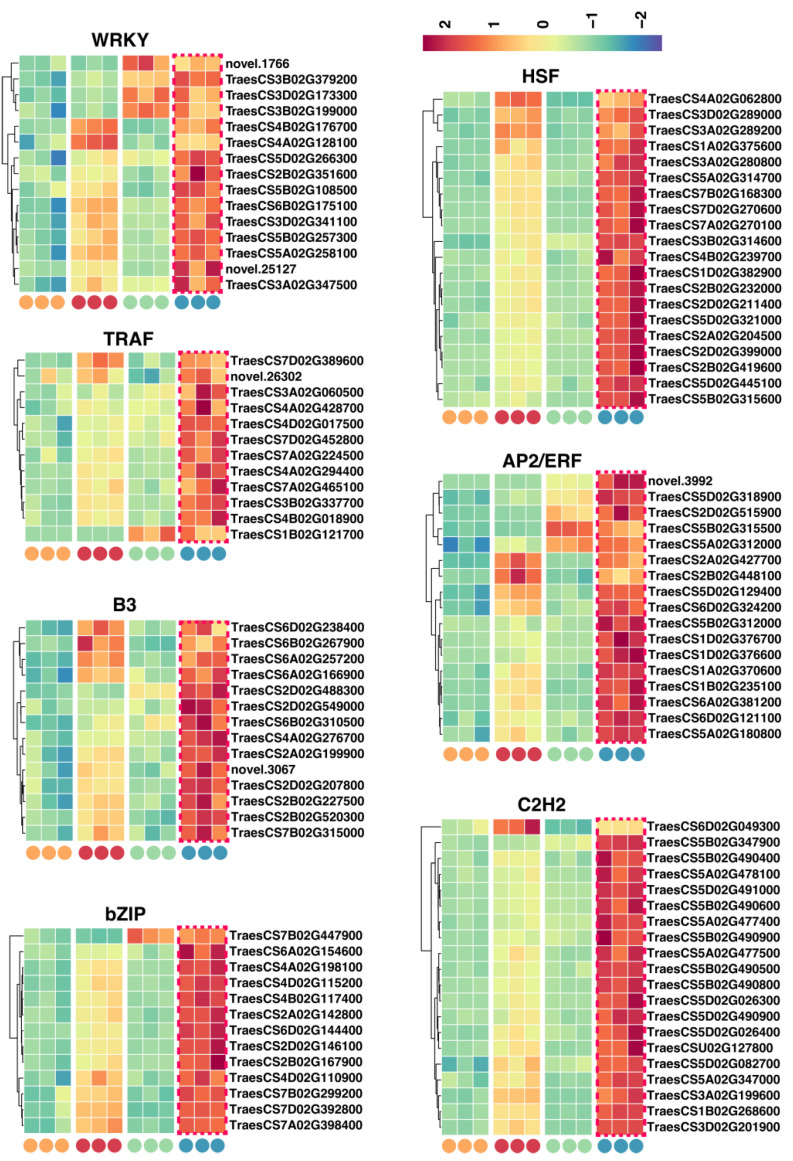
Expression profiling of transcription factors (TFs) that were negative regulators of wheat seed vigor.

TFs play crucial roles throughout the development, maturation, and aging of plant seeds. Hence, we selected TFs associated with seed vigor during the seed aging process for further analysis. The results indicated that WRKY, TRAF, C3H, bZIP, HSF, AP2/ERF, and C2H2 might be key negative regulators of seed vigor in the aging process of LT seeds. We also identified 111 genes that were most significantly upregulated specifically in the LT 48 h group: 15 from WRKY, 12 from TRAF, 14 from C3H, 13 from bZIP, 20 from HSF, 17 from AP2/ERF, and 20 from C2H2 (Figure 8). These TF genes may be potential negative regulators of wheat seed vigor. Their high expression could lead to reduced vigor in LT wheat seeds.

**Figure 9:**
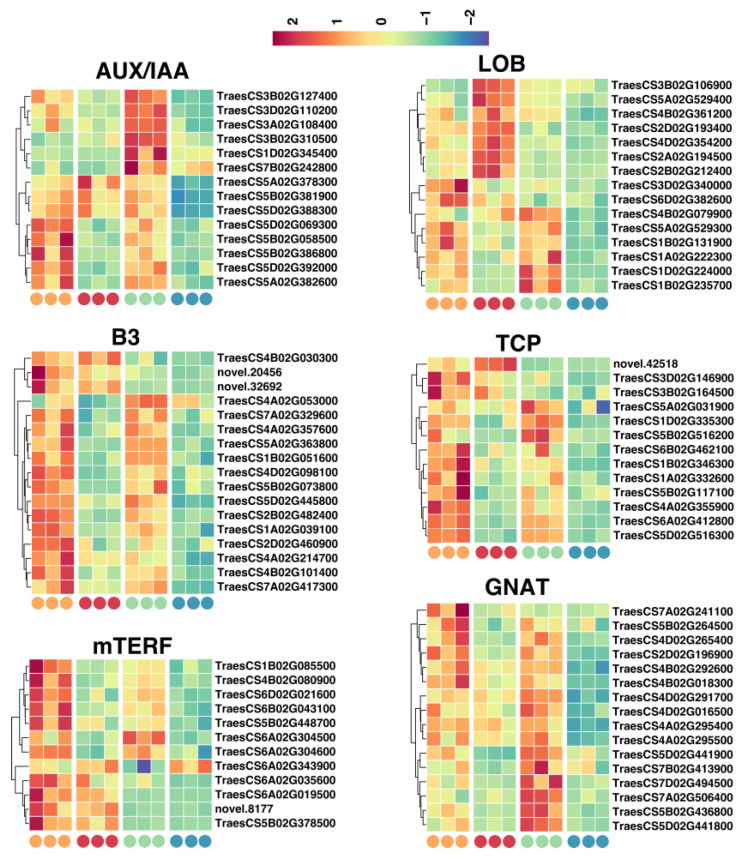
Expression profiling of TFs that were positive regulators of wheat seed vigor.

Conversely, our analysis unveiled that AUX/IAA, B3, mTERF, LOB, TCP, and GNAT might be pivotal positive regulators of vigor in the aging process of LT seeds. We identified the most significant genes from the above TF families: 14 from AUX/IAA, 17 from B3, 11 from mTERF, 14 from LOB, 13 from TCP, and 16 from GNAT, all of which were specifically downregulated in the LT 48 h group (Figure 9). We thus categorized these 85 TF genes as potential positive regulators of wheat seed vigor, the low expression of which may ultimately result in reduced vigor during the aging of LT wheat seeds. In conclusion, the orchestrated modulation of these identified TFs, acting as either positive or negative regulators, may play vital roles in dictating the vitality of wheat seeds throughout aging, presenting insightful perspectives for in-depth exploration of the intricate molecular mechanisms underlying seed longevity and vigor in cereal crops.

## 3. Discussion

Plant metabolomics has been widely applied to the investigation of patterns of metabolite accumulation and its underlying genetic basis via the identification of genes involved in metabolism, which is currently a topic of interest in modern plant biology [22,23,24]. As the final products of genome expression, metabolites directly determine the biochemical characteristics of a cell or tissue [25], which allows metabolomic data to be used to explain the biochemical mechanisms underlying artificial seed aging between wheat cultivars. Meanwhile, integrated transcriptomic and metabolomic analysis allows for the more precise representation of gene-to-metabolite networks [26,27,28] and, thus, is an effective method for deciphering the mechanisms involved in artificial seed aging. In this study, we combined transcriptomic and metabolomic analyses to generate dynamic maps of artificial aging processes involving wheat seeds, aiming to better understand the physiological mechanisms at the molecular and biochemical levels.

In this study, PCA and hierarchical cluster analysis (HCA) were performed to assess the accumulation patterns of metabolites among seed samples. We noticed a common trend of changes in wheat metabolite accumulation between cultivars, indicating that artificial aging may produce the same alterations in different wheat cultivars.

To investigate the specific accumulation of DIMs by two differentiating factors—artificial aging and intercultivar differences—Venn diagrams were generated for deeper analysis. The results showed that more organic acids (DL-3-phenyllactic acid, ethylmalonic acid, glutaric acid, oxalic acid, etc.) were upregulated in the process of artificial aging. The contents of organic acids (3-methylmalic acid, citric acid, isocitric acid, quinic acid, etc.) in the HT cultivar were lower than those in the LT cultivar, while the contents of amino acids and their derivatives (5-oxo-L-proline, L-cystathionine, L-glutamine-O-glycoside, Lys-Tyr, etc.) were higher. Organic acids accumulated during artificial aging in the LT cultivar may play a key role in the aging process, and may be crucial in regulating seed vitality. In addition, DIMs and DEGs were enriched in pathways related to carbohydrates, nucleotides, and other metabolites by the artificial aging process.

Lipid peroxidation has been proven to cause a loss of seed viability [28,29,30,31]. Lipid peroxidation products are always detected in seeds after artificially accelerated aging at high temperature and humidity levels, but are rarely detected in the early degradation process of naturally aged seeds. This may be due to the death of some seeds from accelerated aging, leading to the accumulation of lipid peroxidation products [29,30]. Protein and sugar are the two main storage substances in seeds, with sugar being the most important respiratory substrate and source of nourishment and energy for the growth and development of seed embryos [31,32]. During seed storage, soluble sugar is continuously decomposed by respiration, resulting in a decrease in the total sugar content with the extension of storage time [33]. In this study, pathways related to organic acid metabolism and carbohydrates were significantly enriched with DIMs and DEGs caused by artificial aging. Related pathways were detected, such as glycolysis/gluconeogenesis, pyruvate metabolism, and glyoxylate and dicarboxylate metabolism. Protein is the product of gene expression, and changes in it reflect changes in genetic stability during the seed aging process [34]. Seed storage proteins (SSPs) provide nitrogen for germination and seedling growth, and are closely related to the formation and maintenance of seed vitality [35]. The combined metabolome and transcriptome analysis of cultivar differences showed significant enrichment in some carbohydrate and amino acid pathways. Related pathways were identified, such as cysteine and methionine metabolism, glutathione metabolism, amino sugar, and nucleotide sugar metabolism.

Some of the significant DEGs in the aforementioned related pathways may play crucial roles. The role of *ACS* in eliminating fermentative intermediates is supported by an enhanced sensitivity of the acs1 mutant to exogenous acetate, ethanol, and acetaldehyde [36]. Interestingly, acetate is able to support high rates of plant growth, but this growth is blocked by the acs1 mutant [37]. A study in Arabidopsis found that *F16P2* plays an important role in aging [38]. Map-based cloning and a complementation test showed that the floury endosperm phenotype was defined by a gene of *OsPPDKB*, which encodes pyruvate orthophosphate dikinase (PPDK, EC 2.7.9.1) [39]. It was also found that the gene was highly expressed in endosperm, and PPDK played an important role in starch metabolism and structure in rice endosperm [40]. These genes may influence changes in the metabolites of organic acids such as lactic acid. However, *BCAT3*, *CHI1*, *GAUT1*, and *GAUT4* play important roles in the metabolism of amino acids, glucosides, ketones, and galactose [41,42], which may cause the difference in seed vitality.

During the germination of aging seeds, the participation of transcription factors can ensure that the target gene is expressed at a specific time in a specific space. In Arabidopsis (*Arabidopsis thaliana* L.), the transcription factor AHL4 interacts with phospholipid acid to regulate the rate of seed germination, the length of main root after seed germination, the rate of lipid degradation during seed germination, and seedling formation, thus affecting seedling formation [21]. In wheat, the transcription factor TRIHELIX plays an important role in regulating seed vigor after aging [43]. Our study found that WRKY, TRAF, C3H, Bzip, HSF, AP2ERF, and C2H2 may be key negative regulators of Nakatane viability during LT seed aging, while AFSIAA, B3, MTERF, LOB, TCP, and gnat may be key positive regulators of seed vigor during seed senescence. The coordinated regulation of these transcription factors, identified as positive or negative regulators, may play an important role in determining the viability of wheat seeds throughout the aging process.

By interactively comparing metabolomic and transcriptomic data, we identified a series of potential metabolites and corresponding DEGs resulting from artificial aging of wheat seeds and intercultivar vigor differences. These findings provide new insights into the underlying molecular and biochemical basis of the artificial aging of wheat seeds and intercultivar vigor differences.

## 4. Materials and Methods

### 4.1. Wheat Seed Materials and Treatment Conditions 

**Figure 10:**
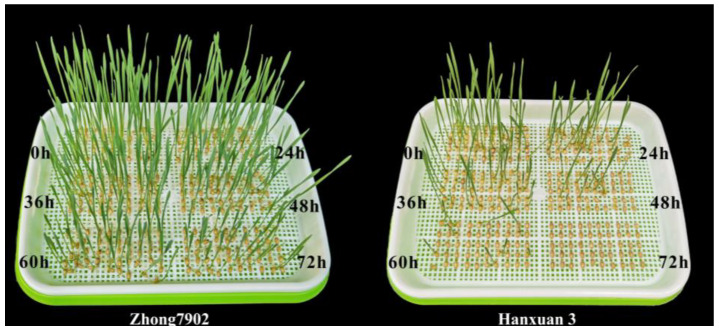
Germination of two wheat varieties with different seed vigor after artificial aging treatment.

Based on the germination rate obtained in preliminary experiments, wheat seeds of two extreme cultivars were selected: “Zhong 7902” for the high-vigor group (HT) and “Hanxuan 3” for the low-vigor group (LT). Accelerated aging was carried out by following the method of a previous study with some modifications [44]. The seeds were subjected to artificial aging treatment at 48 °C with a humidity level of 95% in an aging chamber for different durations: 0 h, 24 h, 36 h, 48 h, 60 h, and 72 h. Afterwards, natural drying of the seeds was performed, followed by a standard germination test. The shortest protruding part of the seed radicle was the same as the length of the seed, which was regarded as normal germination. According to the results of the germination test, wheat seeds aged for 48 h exhibited the greatest disparity between the two cultivars, and these were selected((Figure 10). Four groups were, therefore, prepared for the following transcriptomic and metabolomic analyses: HT seeds without aging (HT 0 h), HT seeds aged for 48 h (HT 48 h), LT seeds without aging (LT 0 h), and LT seeds aged for 48 h (LT 48 h). Three independent biological repeats were performed for each group.

### 4.2. Sample Preparation and Extraction for Widely Targeted Metabolic Profiling

Samples were freeze-dried using a Scientz-100F freeze dryer (Scientz, Ningbo, China) and crushed using a mixer mill (MM 400, Retsch, Düsseldorf, Germany) for 1.5 min at 30 Hz. Fifty milligrams of each sample were dissolved in 1.2 mL of 70% methanol, followed by vortexing for 30 s every 30 min, and this was repeated six times. After centrifugation at 12,000 rpm for 3 min, supernatants were collected and filtered using a membrane with a 0.22 μm pore size. Then, samples were ready for ultra-performance liquid chromatography-tandem mass spectrometry (UPLC-MS/MS) analysis.

### 4.3. UPLC-MS/MS Conditions

UPLC-MS/MS-based, widely targeted metabolome analysis was performed on an Applied Biosystems 4500 QTRAP system (Applied Biosystem Sciex, Thornhill, Ontario, Canada), which was equipped with an Agilent SB-C18 column (1.8 μm, 2.1 mm × 100 mm). The mobile phase A consisted of ultra-pure water (containing 0.1% formic acid), while phase B used 1% formic acid in acetonitrile. The elution gradient was initiated from phase B at 5% and increased linearly to 95% within 9 min, followed by maintenance for 1 min at 95%. Phase B then decreased to 5% in 1 min, and was maintained at 5% for 3 min. The flow rate was set at 0.35 mL/min, with a column temperature of 40 °C and an injection volume of 4 μL.

Further, the temperature for electrospray ionization was 550 °C. The ion source parameters were an ion spray voltage of +5500 V/−4500 V; curtain gas with 25 psi; ion source gas (GS) 1 and 2 with 50 and 60 psi, respectively; and collision-induced dissociation at high energy levels. The QqQ scan was operated in multiple-reaction monitoring (SRM) mode, and collision gas (helium) at medium. Parameters such as declustering potential (DP) and collision energy (CE) were optimized for each specific MRM ion pair. Ion pairs were monitored based on the metabolites eluted during the period. Based on the MWDB database (metware database), the compounds were identified according to second-order spectral information. The determination metabolites was completed by multiple reaction monitoring (MRM) of triple quadrupole mass spectrometry.

### 4.4. Transciptome Analysis

Total RNA isolation and extraction were performed as described previously, with some modifications [45]. Libraries were constructed using the NEBNext Ultra RNA Library Prep Kit for Illumina (NEB, Ipswich, MA, USA). Qualified transcriptome libraries were paired-end (150 bp) sequenced on the Illumina HiSeq6000 sequencing platform (Illumina, USA), after which raw paired-end RNA-Seq reads were filtered into clean data using FASTP (version 0.19.5) [46]. RNA clean reads were aligned to the reference genome (TGACv1, https://plants.ensembl.org/Triticum_aestivum/Info/Index, accessed on 11 October 2023) using HISAT2 (version 2.0.4) [47]. The FPKM (fragments per kilobase of transcript per million fragments mapped) value was calculated as the gene expression level. DESeq2 (version 1.10.1), with a model based on the negative binomial distribution, was used to identify differentially expressed genes (DEGs) [48], and the screening threshold was set at padj < 0.05 and log2 (foldchange) ≥ 1. The data sets were submitted to the SRA database (accession number: SUB14113019).

### 4.5. Statistical Analysis

The metabolites were extracted by the intelligent secondary spectral matching method, which was independently constructed using the plant metabolite database (www.metware.cn/ accessed on 11 October 2023) of Wuhan Metwell Company, and the relative content of metabolites was detected using the MRM detection mode. Data obtained from metabolic profiling were normalized for principal component analysis (PCA) and partial least squares-discriminant analysis (PLS-DA) [49,50], The mass spectrometry data were processed using software Analyst 1.6.3 to qualitatively analyze the metabolites of the samples. Metabolites were considered to be significantly changed in content when the variable importance in project (VIP) was ≥1 and the fold change was ≥2 or ≤ 0.5. Fisher’s exact test was applied to identify significant KEGG pathways with a false discovery rate (FDR) < 0.05 [51]. Gene–metabolite pairs with Pearson’s correlation coefficients > 0.8 in pathways associated with seed aging between cultivars were used to construct the transcript–metabolite network.

### 4.6. Quantitative Real-Time Polymerase Chain Reaction (q-PCR) Analysis

q-PCR analysis was performed using an SYBR Green system (TaKaRa, Dalian, China). PCR amplications were carried out using the qTOWER 2.2 system (Analytik Jena, Germany). The cycling conditions were 3 min of predenaturation at 95 °C, then 95 °C for 10 s, 60 °C for 30 s, and 72 °C for 90 s (40 cycles). The housekeeping gene *Actin* was used as an internal control to calculate relative gene expression. Gene expression for each sample was measured by q-PCR using the relative quantification method [52]. The primers were designed using Primer Premier 6.0 (Premier Biosoft International, Palo Alto, CA, USA) and are listed in Supplementary Table S3. The same procedure was performed across all three independent biological and technical repeats. Primers were designed based on the Ensembl Plants database (http://plants.ensembl.org, accessed on 11 October 2023).

## 5. Conclusions

In this study, combining metabolic and transcriptomic analyses, wheat seeds of two cultivars with different vigor were analyzed as they underwent artificial aging, and the physiological mechanism of seed aging was preliminarily revealed. Metabonomic analysis showed that artificial aging had an important effect on acetaldehyde and dicarboxylic acid metabolism, biosynthesis of secondary metabolites, and fatty acid degradation in wheat seeds. Metabolic pathways were mainly affected by high- and low-vigor varieties. Through transcriptome analysis, it was found that the expression of key genes such as ACS, F16P2, and PPDK1 was significantly affected by artificial aging. Transcription factors WRKY, TRAF, C3H, bZIP, HSF, AP2/ERF, and C2H2 may be the key negative regulators of seed viability during seed aging, while AUX/IAA, B3, mTERF, LOB, TCP, and GNAT may be key positive regulators of seed vitality during seed aging. Glycolysis/ gluconeogenesis, pyruvate metabolism, acetaldehyde, and dicarboxylic acid metabolic pathways play important roles in artificial aging. In addition, the pathways related to the metabolism of amino acids and peptides affect the vigor of wheat seeds. BCAT3, CHI1, GAUT1, and GAUT4 may be the key genes that contribute to the vigor differences among wheat varieties. In summary, this study clarified the mechanism of wheat seed aging and vigor differences at the genetic and metabolic levels, therefore providing a possible scheme for the storage of germplasm resources and the improvement of agricultural production.

## Data Availability

The transcriptomic sequencing data can be downloaded from the SRA database (accession number: SUB14113019).

## Supplementary Materials

The following supporting information can be downloaded at: https://www.mdpi.com/article/10.3390/ijms25010526/s1.
