# Supplementary material for: Integrated Transcriptomic and Metabolomic Analyses Identify Critical Genes and Metabolites Associated with Seed Vigor of Common Wheat
Source: Int J Mol Sci. 2023 Dec 30;25(1):526. doi: 10.3390/ijms25010526 (PMC10779259; doi:10.3390/ijms25010526)
Supplement: Supplementary file 1 [file ijms-25-00526-s001.zip › Supplementary Materials.pdf]

Supplementary Figure S1. The gene expression pattern of the transcriptome was verified by qRT-PCR.

Supplementary Figure S2. KEGG enrichment and the expression profile across DAMs and DEGs.
