# Supplementary material for: Integrated Transcriptomic and Metabolomic Analyses Identify Critical Genes and Metabolites Associated with Seed Vigor of Common Wheat
Source: Int J Mol Sci. 2023 Dec 30;25(1):526. doi: 10.3390/ijms25010526 (PMC10779259; doi:10.3390/ijms25010526)
Supplement: Supplementary file 1 [file ijms-25-00526-s001.zip › Supplementary Table S3 The primer sequence of qRT.pdf]

Supplementary Table S3 The primer sequence of qRT-PCR

Gene name	Gene ID	Primer	Sequence (5'to3')
	TraesCS2A02G423500	423500-F	AGGGTGTATCTTCTGCTGCG
		423500-R	CGGAAAGAGGTCCACCACAA
G6PI	TraesCS1D02G041200	041200-F	CATAAAAAGCCAGCAGCCCG
		041200-R	TTCCATAGGCAAGCGCATCA
IDH	TraesCS3D02G175100	175100-F	GCAGGTTTGAGGATGCAGGA
		175100-R	GCAAGCCCAAACATAGCCAC
PGD	TraesCS7D02G030000	030000-F	CCCTGCATCCTTCGTCAACT
		030000-R	TCAACGATGCAGTCACCCTG
GPX	TraesCS6A02G246400	246400-F	CCTTGCTTTCCCATGCAACC
		246400-R	GCGAGTGCAAGCAAACTGAA
GR	TraesCS1A02G045700	045700-F	GATGGTTCGGAGAAGGCACT
		045700-R	TTCGCCCCATTGATGTAGGG
GOLS	TraesCS4B02G179300	179300-F	TCATGGACTGCTTCTGCGAG
		179300-R	CGTCACCTTTTCGGGACACT
USP	TraesCS7D02G412100	412100-F	GGCAAAGGAAGCCATTGGTG
		412100-R	CAGTTGCACGAAGGAGTGGA
GALK	TraesCS5B02G510900	510900-F	GCAACCGATGTGCAACTACC
		510900-R	CGAGAACAATCGCTGCCAAG
INV	TraesCS4B02G356800	356800-F	TTCGGTGGTTGAGAGCTTCG
		356800-R	TATCGGTCACCCCGTTGTTG
XCP1	TraesCS2B02G576300	576300-F	ACTACTGGATCGTCCGCAAC
		576300-R	TCCCACTGGTCACGTTGATG
ARF	TraesCS2B02G578500	578500-F	ATGCAGTCGCCTCAAGAACA
		578500-R	CAGTTTGCTGTTGGCTTGCT
Actin	TaActin	TaActin-F	TGTTGTTCTCAGTGGAGGTTCT
		TaActin-R	CTGTATTTCCTTTCAGGTGGTG
