# Supplementary figures and images for: Integrated Transcriptomic and Metabolomic Analyses Identify Critical Genes and Metabolites Associated with Seed Vigor of Common Wheat
Source: Int J Mol Sci. 2023 Dec 30;25(1):526. doi: 10.3390/ijms25010526 (PMC10779259; doi:10.3390/ijms25010526)

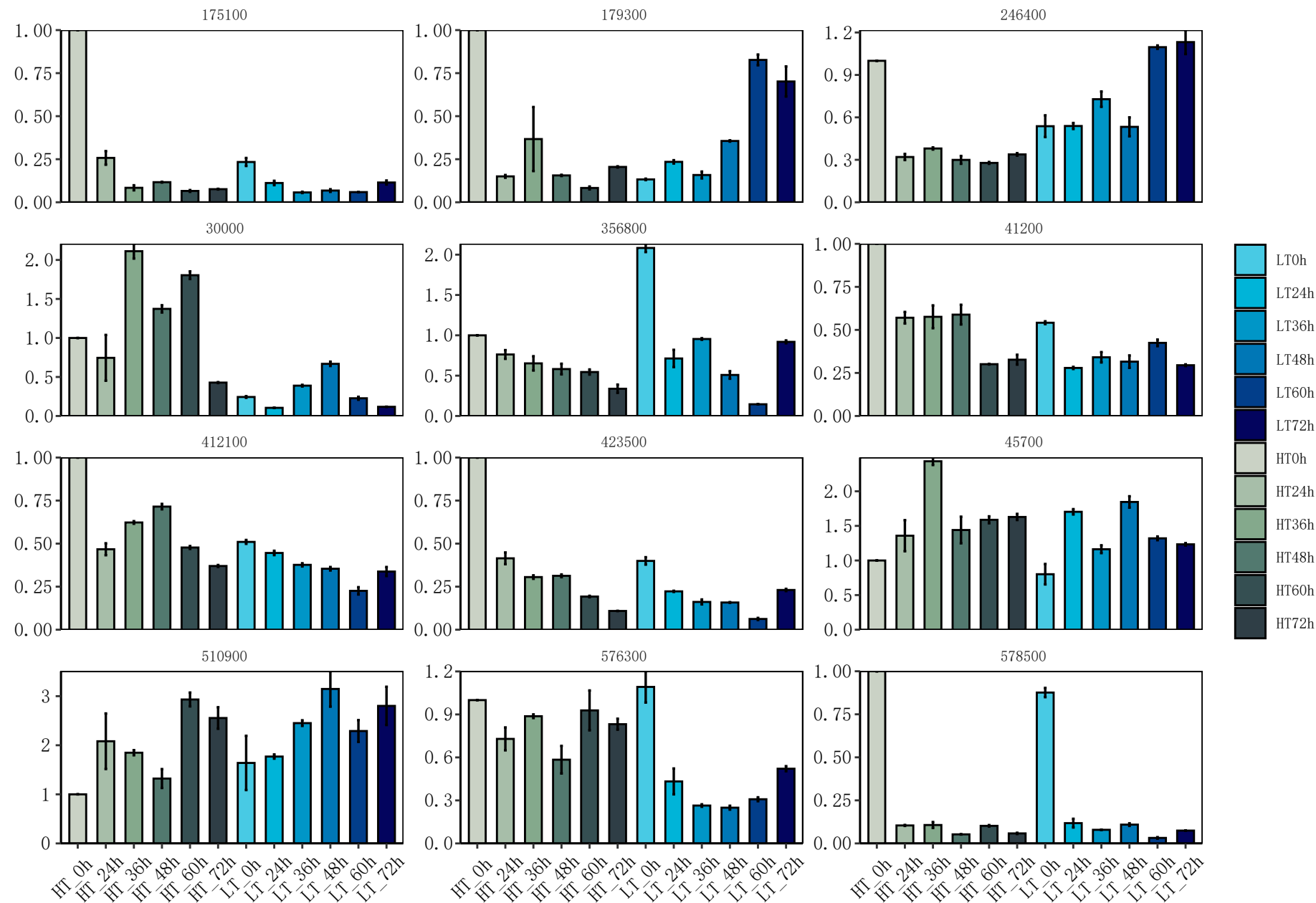

Supplement: Supplementary file 1 [file ijms-25-00526-s001.zip › Figure S1.pdf]

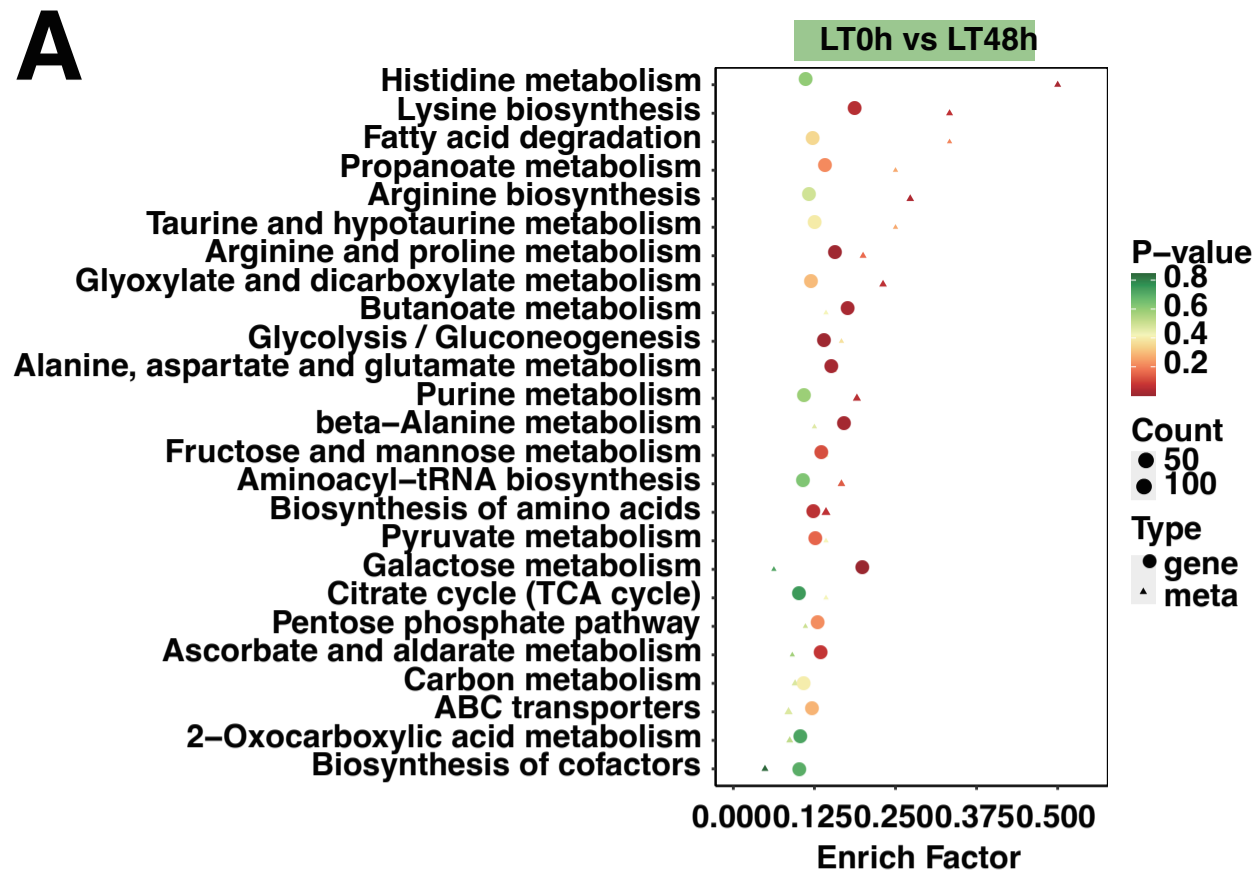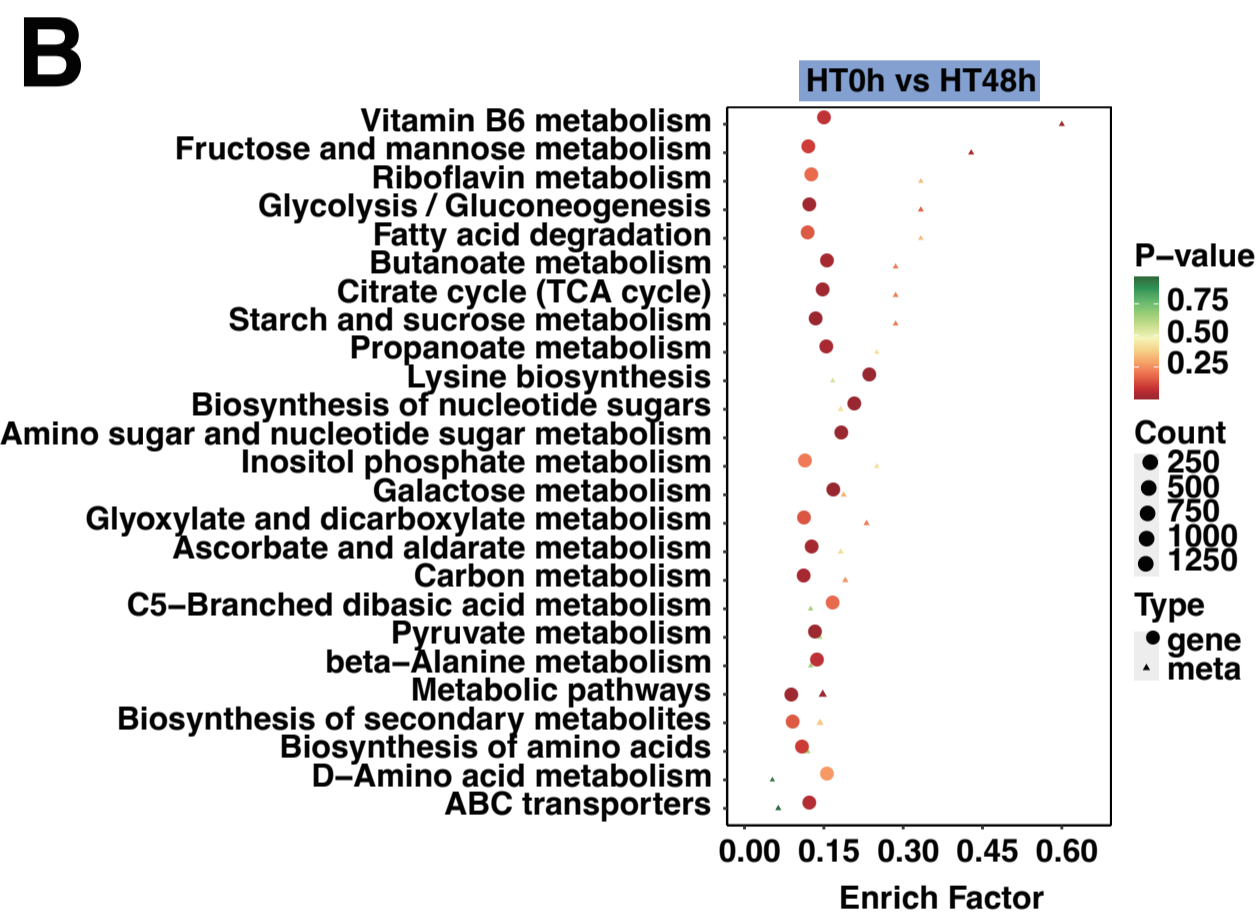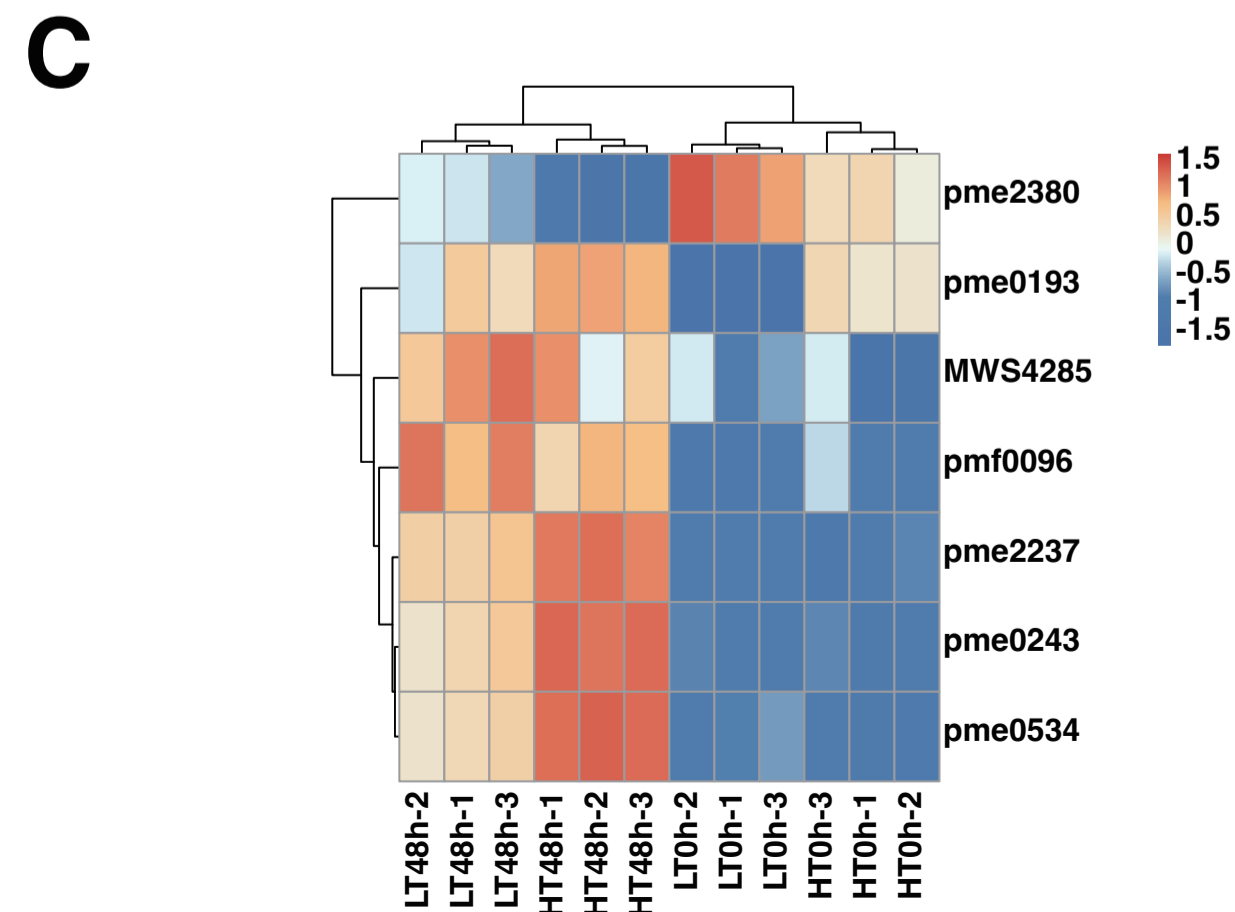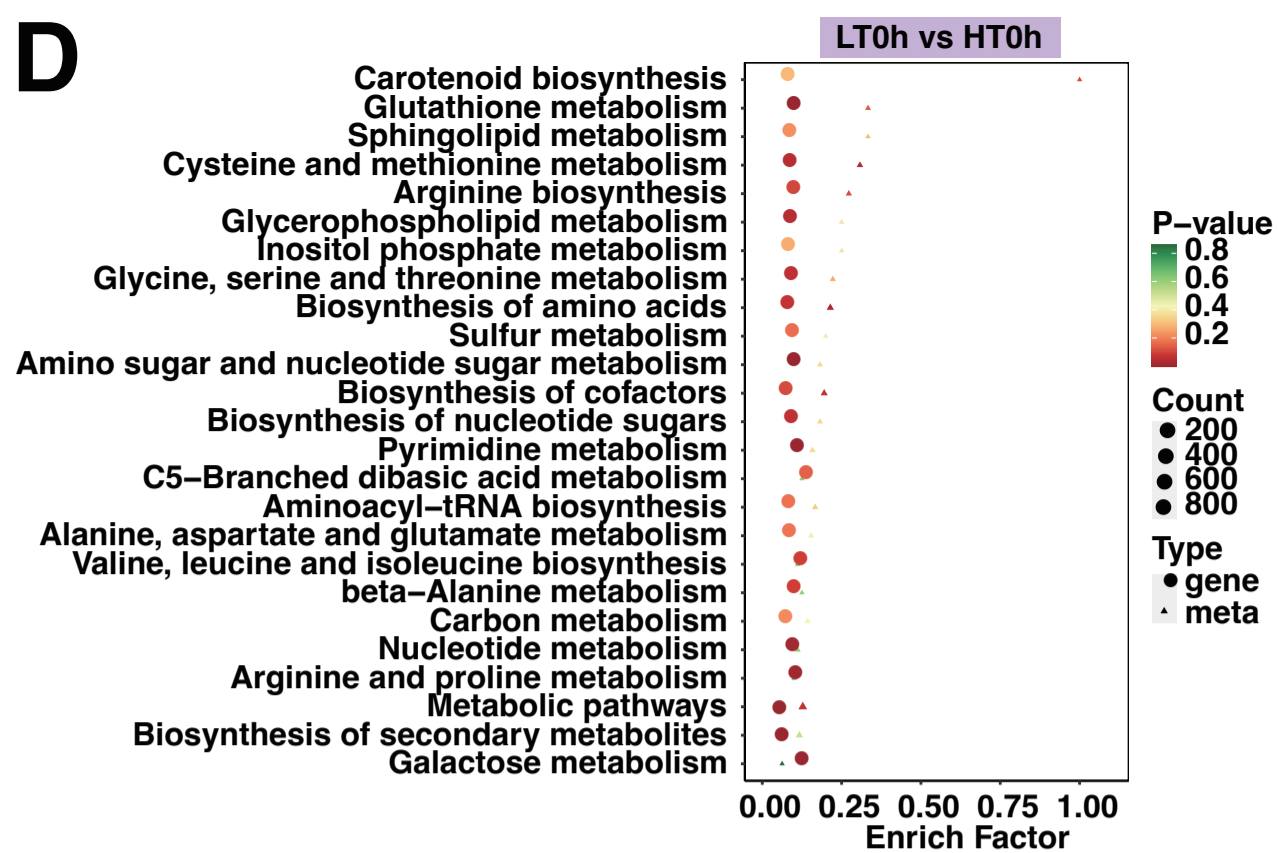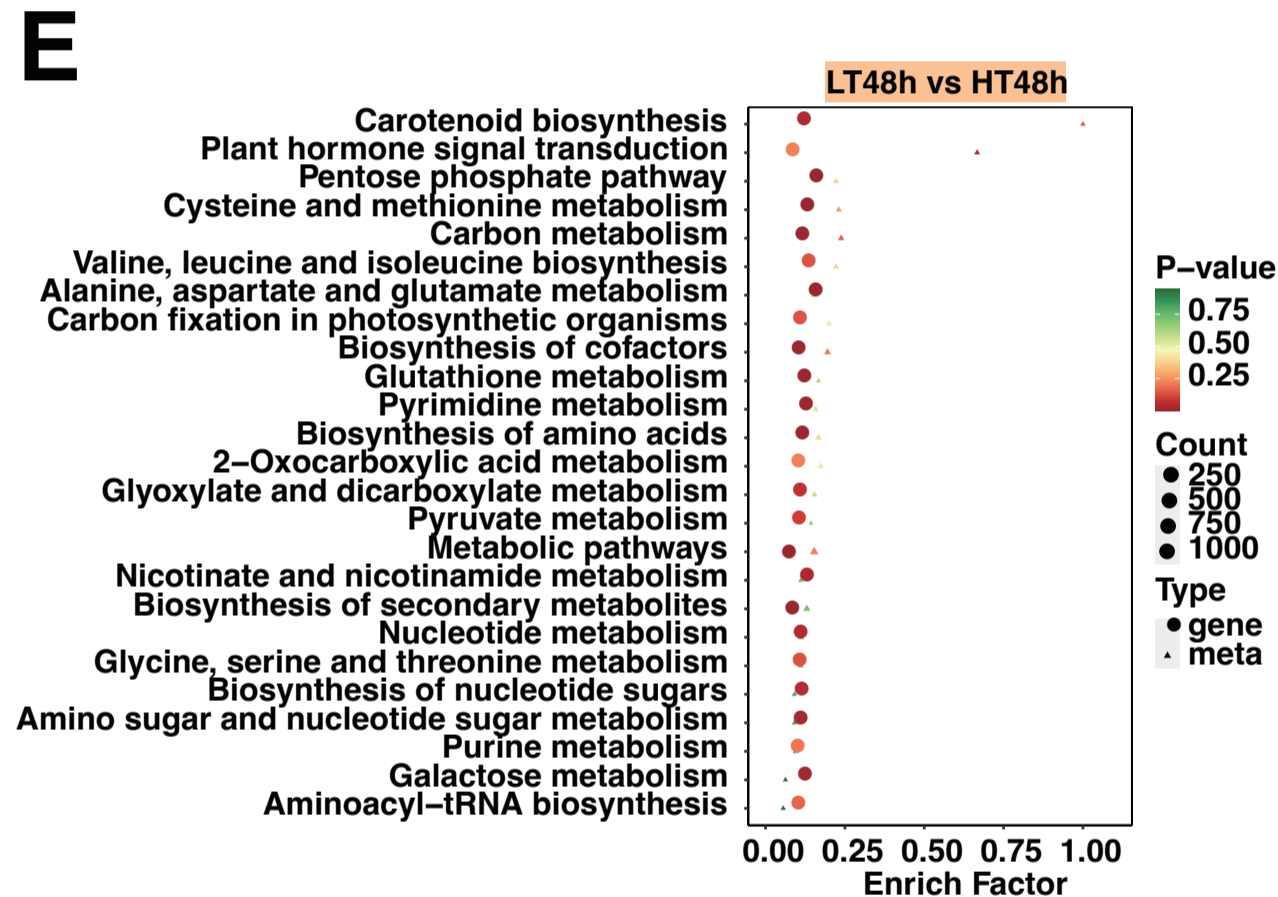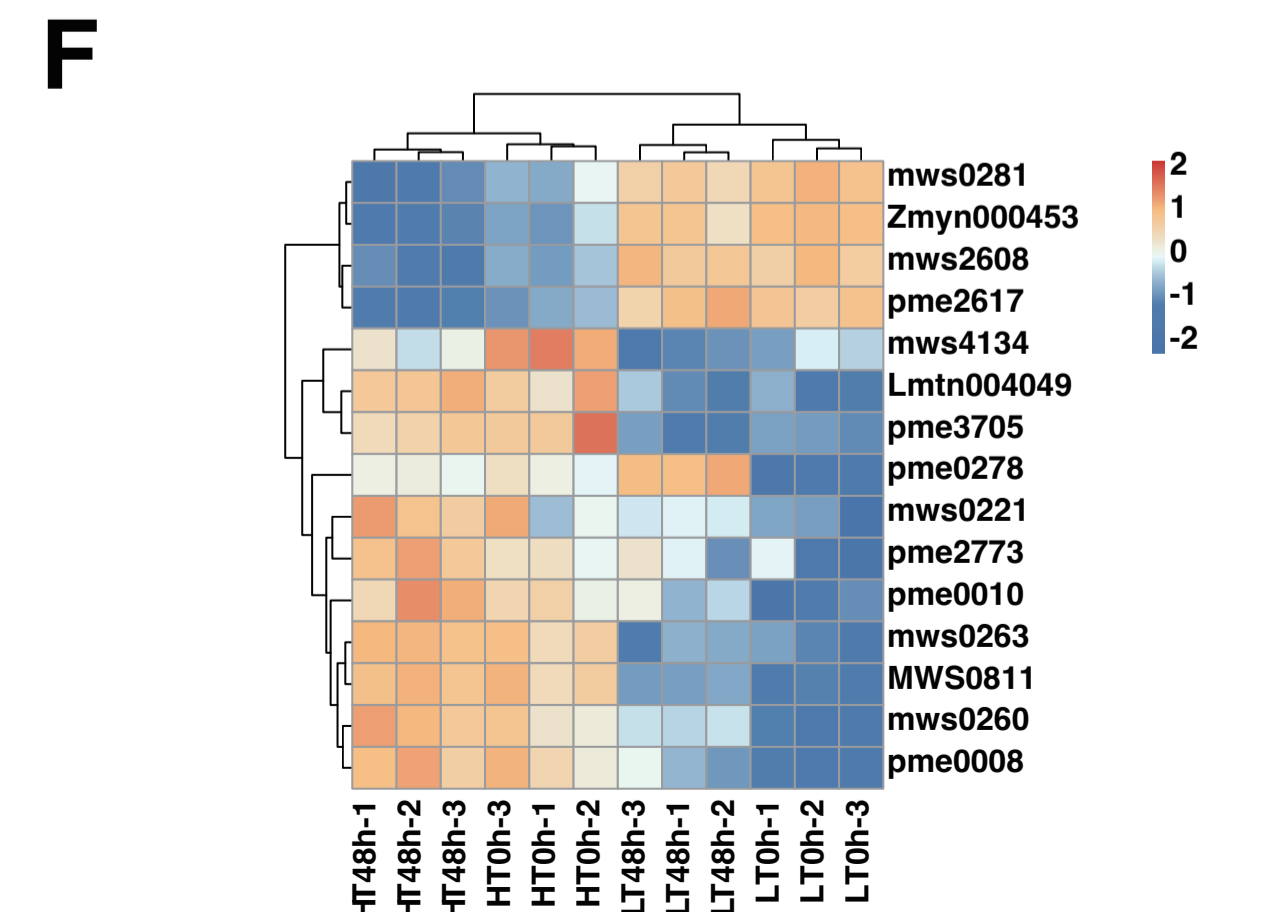

Supplement: Supplementary file 1 [file ijms-25-00526-s001.zip › Figure S2.pdf]
